# Targeted disruption of the orphan receptor Gpr151 does not alter pain-related behaviour despite a strong induction in dorsal root ganglion expression in a model of neuropathic pain

**DOI:** 10.1016/j.mcn.2016.11.010

**Published:** 2017-01

**Authors:** Fiona E. Holmes, Niall Kerr, Ying-Ju Chen, Penny Vanderplank, Craig A. McArdle, David Wynick

**Affiliations:** a School of Physiology, Pharmacology & Neuroscience, Medical Sciences Building, University Walk, Bristol BS8 1TD, UK; b School of Clinical Sciences, Dorothy Hodgkin Building, Whitson Street, Bristol BS1 3NY, UK; c School of Clinical Sciences, Medical Sciences Building, University Walk, Bristol BS8 1TD, UK

**Keywords:** Gpr151, Galanin, GalR1, GalR2, Dorsal root ganglion, Pain

## Abstract

**Background:**

Gpr151 is an orphan GPCR whose function is unknown. The restricted pattern of neuronal expression in the habenula, dorsal horn of the spinal cord and dorsal root ganglion plus homology with the galanin family of receptors imply a role in nociception.

**Results:**

Real-time quantitative RT-PCR demonstrated a 49.9 ± 2.9 fold highly significant (*P* < 0.001) increase in Gpr151 mRNA expression in the dorsal root ganglion 7 days after the spared nerve injury model of neuropathic pain. Measures of acute, inflammatory and neuropathic pain behaviours were not significantly different using separate groups of Gpr151 loss-of-function mutant mice and wild-type controls. Galanin at concentrations between 100 nM and 10 μM did not induce calcium signalling responses in ND7/23 cells transfected with Gpr151.

**Conclusions:**

Our results indicate that despite the very large upregulation in the DRG after a nerve injury model of neuropathic pain, the Gpr151 orphan receptor does not appear to be involved in the modulation of pain-related behaviours. Further, galanin is unlikely to be an endogenous ligand for Gpr151.

## Highlights

• Gpr151 is an orphan GPCR of unknown function.
• RT-PCR demonstrated a 50-fold increase in Gpr151 mRNA in the DRG 7 days after a nerve injury model of neuropathic pain.
• Acute, inflammatory and neuropathic pain behaviours were unchanged between Gpr151 mutant mice and wild-type controls.
• Galanin did not induce calcium signalling responses in ND7/23 cells transfected with Gpr151.

## 1 Background

An increasing number of groups have used microarray analysis of mRNA transcripts to identify the molecular changes in the dorsal root ganglia (DRG) and spinal cord that occur after peripheral nerve injury and in models of neuropathic pain (Costigan et al., 2002, Griffin et al., 2003). The aim of these studies is to identify genes that are up- or down-regulated after nerve injury in the expectation that some may be involved in the development and/or maintenance of neuropathic pain and would thus be targets for analgesic drug development (Costigan et al., 2002, Griffin et al., 2003).

The orphan G-protein coupled receptor (GPCR) Gpr151 is a member of the class A rhodopsin-like family and has recently been shown to be significantly up-regulated in the DRG following neuronal injury models of neuropathic pain (Jiang et al., 2015, Reinhold et al., 2015). Gpr151 was originally identified as having homology to galanin receptors, and variously designated as GPCR10, GPCR-2037, GalRL (Berthold et al., 2003, Ignatov et al., 2004, Takeda et al., 2002) or PGR7 (Vassilatis et al., 2003). Sequence-structure phylogenetic analysis of the rhodopsin-like family placed Gpr151 within ‘cluster 14’, together with galanin receptor 1 (GalR1), GalR2, GalR3 and the kisspeptin receptor (Kiss1R/Gpr54) (Kakarala and Jamil, 2014), and comparable sequence analysis placed Gpr151 within the wider ‘SOG’ subfamily that includes somatostatin, opioid, galanin and kisspeptin receptors (Civelli et al., 2013), though other reports mention an ambiguous relationship to other rhodopsin-like family members (Bjarnadottir et al., 2006, Gloriam et al., 2007, Vassilatis et al., 2003).

Strong expression of Gpr151 mRNA within the brain is restricted to the medial and lateral habenulae (Berthold et al., 2003, Ignatov et al., 2004), and is also detected in the paraventricular nucleus of the thalamus ((Ignatov et al., 2004, Lein et al., 2007) http://www.brainatlas.org). The medial habenula and lateral habenula have distinct neural connectivity, and play roles in pain, analgesia, stress, memory, depression and nicotine withdrawal (Hikosaka, 2010, Shelton et al., 2012a), and Gpr151 is among the most enriched transcripts within the habenula (Quina et al., 2009). In the peripheral nervous system Gpr151 is expressed in the DRG, trigeminal ganglia and vestibular ganglia (Ignatov et al., 2004). Although the endogenous ligand for Gpr151 is unknown, it has been suggested to share structural features with galanin due to a weak activation by galanin (EC_50_ of 2 μM), but not by other peptides including galanin 1–16 or galanin-like peptide (Ignatov et al., 2004).

In this study we have focused on the expression of Gpr151 in the DRG and demonstrated a marked increase in mRNA levels after nerve injury, in contrast to the decrease in expression of GalR1 and GalR2. Comparing mice homozygous for a targeted disruption of Gpr151 (MUT) to strain-matched wild-type (WT) controls, no significant differences were observed in a range of pain behaviours in intact animals or in models of inflammatory or neuropathic pain. Further, we show that the receptor is not activated by galanin, consistent with its status of an orphan receptor.

## 2 Results

### 2.1 Gpr151 mRNA is strongly induced after spared nerve injury

**Fig. 1:**
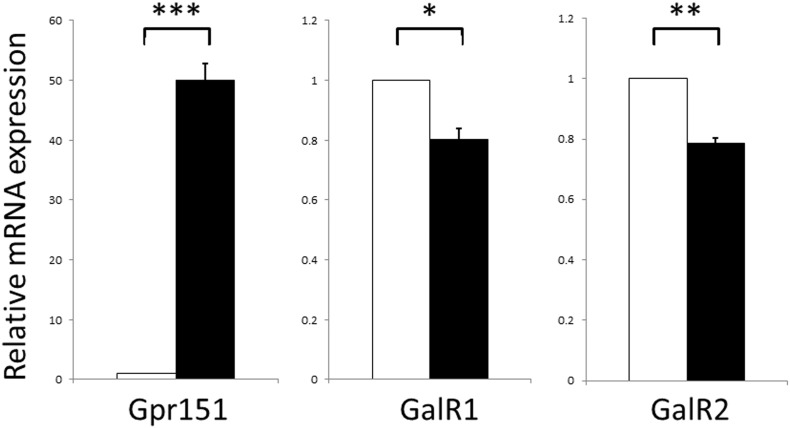
Gpr151 and GalR1/GalR2 mRNA expression levels are differentially affected in adult mouse DRG one week after SNI. In quantitative RT-PCR assays the expression of Gpr151 mRNA increased by 49.923 ± 2.907 fold compared to control (*n* = 4; *P* = 0.0005), whereas GalR1 and GalR2 mRNAs respectively decreased to 0.803 ± 0.037 (*n* = 4; *P* = 0.0131) and 0.786 ± 0.019 (*n* = 4; *P* = 0.0015) of control. Data are shown as means ± SE, in which expression after SNI (filled boxes) was compared with contralateral controls of 1.00 relative units (open boxes). Note the difference in scales between the Gpr151 and GalR1/GalR2 results. *P < 0.05; **P < 0.01; ***P < 0.001.

To test whether the expression of Gpr151 is altered in DRG after the spared nerve injury (SNI) model of neuropathic pain, real-time quantitative RT-PCR was used with expression levels normalized to Gapdh (glyceraldehyde 3-phosphate dehydrogenase) mRNA, which is unchanged following peripheral nerve injury (Kerr et al., 2008). Seven days after SNI, the expression of Gpr151 mRNA increased 49.9 ± 2.9 fold compared with control (*P* < 0.001) in lumbar L4 and L5 DRG (Fig. 1). As the Gpr151 gene is intronless, expression levels could be compromised by DNA contamination. This was not a problem with the samples tested as the average threshold cycle values (C_t_) at which fluorescence exceeded the threshold limit in reverse transcribed (RT +) control RNA reactions was 30.7 cycles, whereas that of non-reverse transcribed (RT-) control RNA was > 40 cycles.

Previously we have detected mRNA expression of the galanin receptors GalR1 and GalR2 in mouse DRG, whereas GalR3 expression is too low for reliable comparison between samples (Hobson et al., 2006). Here we show that seven days after SNI there is a decrease of approximately 20% in the expression of both GalR1 (*P* < 0.05) and GalR2 (*P* < 0.01) mRNAs (Fig. 1).

### 2.2 Targeted disruption of GpR151 does not alter pain-like behaviours

**Fig. 2:**
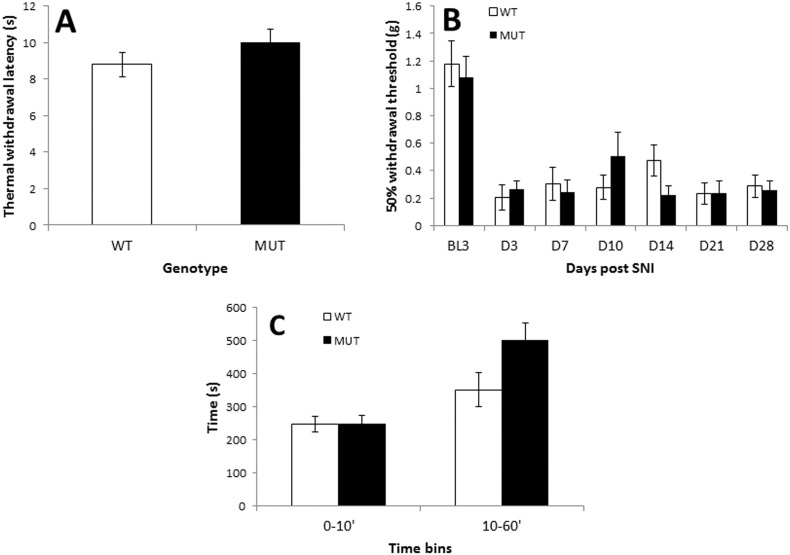
Pain-related behaviours are unchanged in mice lacking Gpr151. A: baseline thermal withdrawal thresholds were similar in WT (open box) and Gpr151 MUT (filled box) mice. B: withdrawal thresholds to mechanical stimulation were tested 3 days before (BL3) and at several time points after spared nerve injury (SNI) for up to 28 days. No differences in pain-related withdrawal thresholds were observed between WT (*n* = 8) and Gpr151 MUT mice (*n* = 10) at any time point. C: mice were injected with 20 μl 1% formalin into the plantar hind paw and nocifensive behaviour monitored in 5 min bins for 1 h. There were no significant differences in pain-related behaviour between WT (*n* = 6) and Gpr151 MUT (*n* = 10) mice in the first (0–10 min) or second (10–60 min) phases of the test.

To investigate whether Gpr151 might play a role in the modulation of acute pain-like behaviour we tested thermal and mechanical withdrawal thresholds in naive WT and Gpr151 MUT mice and found no significant differences between the two genotypes. Thermal withdrawal thresholds were 8.8 ± 0.7 s and 9.9 ± 0.7 s for WT and Gpr151 MUT mice, respectively (Fig. 2A). Mechanical 50% withdrawal thresholds were 1.18 ± 0.17 g and 1.08 ± 0.16 g for WT and Gpr151 MUT mice, respectively (BL3, Fig. 2B). There was also no significant difference in the chronic pain-like allodynia which develops following SNI: by 3 d post-SNI mechanical 50% withdrawal thresholds had decreased to 0.20 ± 0.09 g in the WT and 0.27 ± 0.06 g in the Gpr151 MUT (D3, Fig. 2B). Allodynia was not significantly different between the genotypes at any time point over the 4 weeks following SNI (Fig. 2B). The formalin test of acute inflammatory nociceptive pain showed similar pain-related behavioural responses in both WT and Gpr151 MUT mice over 1 h post-formalin (Fig. 2C). The total amount of time spent in nocifensive behaviours in the first 10 min (due to direct stimulation of nociceptors) was 247 ± 24 s for WT and 248 ± 26 s for Gpr151 MUT; and 351 ± 51 s for WT and 501 ± 51 s for Gpr151 MUT in the second phase (due to inflammatory mechanisms and central sensitisation in the dorsal horn of the spinal cord), which although showing a trend towards greater pain-like behaviour in Gpr151 MUT mice, was not statistically significant (*P* = 0.056).

### 2.3 Gpr151 signalling is not activated by galanin

**Fig. 3:**
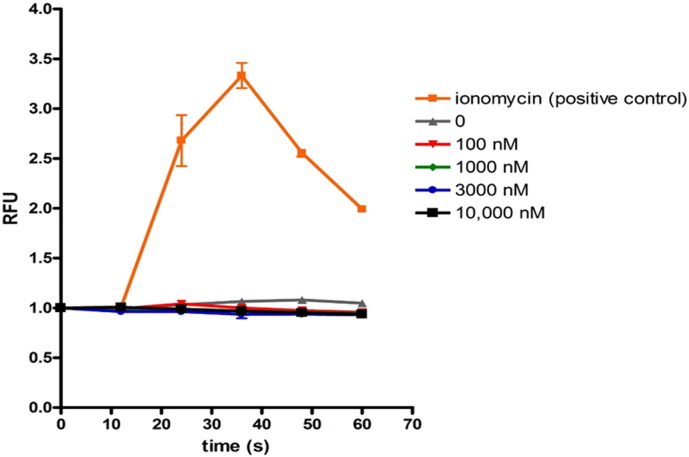
Galanin does not induce calcium signalling in cells expressing Gpr151. ND7/23 cells transfected with Gpr151 were loaded with Fluo-4, exposed to various concentrations of galanin (0–10 μM) or ionomycin as a positive control and calcium signalling measured by an increase in Fluo-4 intensity. RFU = relative fluorescence units. No increase in intracellular calcium was observed at any concentration of galanin in marked contrast to ionomycin.

Since Gpr151 shows homology with the galanin receptors and a previous study had demonstrated weak activation by galanin (EC_50_ of 2 μM), we tested whether galanin could induce intracellular calcium signalling via activation of Gpr151. ND7/23 cells (a hybridoma of mouse neuroblastoma and rat DRG (Wood et al., 1990)) were transiently transfected with Gpr151, and calcium signalling measured using the calcium indicator Fluo-4. As shown in Fig. 3 addition of galanin at concentrations of 100 nM, 1 μM, 3 μM and 10 μM failed to elicit a calcium response indicating that galanin is unlikely to be an endogenous ligand of this orphan GPCR.

## 3 Discussion

Acute pain is a protective adaptive survival response which avoids or reduces potential tissue-damage until healing has occurred (Basbaum et al., 2009, Costigan et al., 2009). In contrast, chronic pain is maladaptive, and is characterised by an enhanced sensitivity to noxious stimuli, as well as pain in response to innocuous or no stimuli (spontaneous pain) which persists beyond a useful defensive and recuperative purpose. Chronic pain remains a significant healthcare problem, is notoriously difficult to treat and there is a huge unmet need for better and more effective analgesic drugs (Costigan et al., 2009, Scholz and Woolf, 2002). The mechanisms underlying the transition from acute to chronic pain remain incompletely understood but it is apparent that changes in both neuronal and non-neuronal cell populations contribute to an overall maladaptive plasticity in the pain matrix (Costigan et al., 2009) that lead to on-going pain states.

Following nerve injury there are significant changes in the transcriptional profiles of cells throughout the pain pathway which are thought to contribute to the development and/or maintenance of chronic pain (Costigan et al., 2002, Griffin et al., 2003). Several groups have used a range of techniques, which include transcriptional profiling and real-time quantitative RT-PCR, to identify and then quantify changes in gene expression in the rodent DRG and/or dorsal horn of the spinal cord after nerve injury models of neuropathic pain (Costigan et al., 2002, Griffin et al., 2003, Jiang et al., 2015, Reinhold et al., 2015). The data described here shows that SNI induces a very clear difference in the mouse DRG 7 days after surgery between the marked 50-fold increase in Gpr151 mRNA and the modest, though significant, 20% decrease in the related receptors GalR1 and GalR2. These changes are comparable to previous reports: Reinhold described a 14.1 fold increase in Gpr151 in mouse DRG 7 days after the chronic constriction model of neuropathic pain (Reinhold et al., 2015) whilst Jiang described a 27.7 fold change in mouse DRG 10 days after the spared nerve ligation model of neuropathic pain (Jiang et al., 2015). The changes in DRG GalR1 and GalR2 mRNA expression are also comparable to previous reports following complete section of the sciatic nerve (axotomy), using in situ hybridization (Sten Shi et al., 1997, Xu et al., 1996) and quantitative RT-PCR (Hobson et al., 2006). Of note, we attempted to perform immunocytochemistry for Gpr151 in the DRG of wildtype mice using a number of different commercially available Gpr151 polyclonal antisera. In all cases identical straining was observed in DRG from wildtype and Gpr151 KO mice strongly implying the observed staining in non-specific, similar to that previously described for other GPCR peptides receptors.

In addition to Gpr151 expression in the DRG, high levels of mRNA and protein have been described in the habenula (Berthold et al., 2003, Broms et al., 2015, Ignatov et al., 2004, Lein et al., 2007) which is located in the dorsal thalamus. The habenula has been implicated in a range of roles that include pain, analgesia, reward and motivational processing (reviewed in (Shelton et al., 2012a, Shelton et al., 2012b)) and is thought to act in part as a relay in descending pain modulation from the nucleus accumbens to the periaqueductal gray (Yu and Han, 1990). Further, peripheral nociceptive stimulation has been shown to excite lateral habenula neurons in both rats and humans (Gao et al., 1996, Shelton et al., 2012a, Shelton et al., 2012b), whilst stimulation of the habenula produces analgesia in the formalin test (Cohen and Melzack, 1986) which is reversed by injection of naloxone onto the habenula (Mahieux and Benabid, 1987).

Since Gpr151: (a) expression is markedly increased in the DRG after models of neuropathic pain; (b) is expressed in the habenula which is modulated by nociceptive stimulation; and (c) is homologous to the galanin receptors which are known to modulate nociception (reviewed in (Lang et al., 2015)), we compared thermal and mechanical nociceptive responses in intact Gpr151 MUT and WT mice and then in models of inflammatory and neuropathic pain. No differences were observed in any modality implying that the receptor does not appear to play a role in nociception. Therefore we propose that Gpr151 and its cognate ligand may play a neuroprotective and/or pro-regenerative role in the DRG after nerve injury, similar to that previously described for galanin and activating transcription factor-3 (Hobson et al., 2006, Holmes et al., 2003, Holmes et al., 2000, Mahoney et al., 2003, Saito and Dahlin, 2008).

The lack of a response with galanin using calcium imaging in ND7/23 cells (a sensory neuron-like cell line) transiently transfected with Gpr151 contradicts, in part, the previous published work by Ignatov (Ignatov et al., 2004). In that paper non-neuronal CHO cells stably transfected with the promiscuous Gα_16_ G-protein and apoaequorin (to allow the use of calcium mobilisation via activation of phospholipase C and inositol trisphosphate) were then transiently transfected with Gpr151 (designated GalRL in that paper). Of note, galanin induced a rise in the calcium-induced bioluminescence response in the vector-transfected cells as well as those transfected with Gpr151, implying the effect of galanin, may in part, be non-specific. Further, the EC_50_ for galanin in the Gpr151 transfected CHO cells was 2 μM which is > 500-fold higher than the reported figures for each of the three galanin receptor subtypes and 20-fold higher than the top of the dose-response curve for each receptor (reviewed in (Lang et al., 2015) and references therein). Taken together, our data using the ND7/23 neuronal cell and the work of Ignatov make it unlikely that galanin is an endogenous ligand for Gpr151. Further, it would seem counter-intuitive for the receptor and ligand to both be very significantly upregulated in the DRG after peripheral nerve injury since that would be likely to cause a rapid desensitisation of any putative function(s) of the Gpr151, in marked contrast to other neuropeptides where a reciprocal relationship in expression between ligand and receptor is often reported (Hokfelt et al., 1994).

## 4 Conclusion

In summary, we found that despite the very large upregulation in the DRG after a nerve injury model of neuropathic pain, the Gpr151 orphan receptor does not appear to be involved in the modulation of pain-related behaviours. Further, galanin is unlikely to be an endogenous ligand for Gpr151. These findings are consistent with the hypothesis that activation of the orphan receptor may instead play a neuroprotective and/or pro-regenerative role in the DRG after nerve injury. Further work is now needed to test the Gpr151 MUT mice in a range of in vitro and in vivo functional and anatomical assays of neuroprotection and regeneration compared to WT controls. However, it is only when the endogenous ligand for the Gpr151 has been identified and characterised that confirmation of these putative roles will be possible.

## 5 Materials and methods

### 5.1 Animals and genotyping

All animals were housed in groups of 2–4 in a 14:10 h light/dark cycle (lights on at 7:00 am) and an ambient temperature of 22 ± 2 °C. Mice had access to standard chow and water ad libitum. Procedures were carried out in accordance with the U.K. Animals (Scientific Procedures) Act 1986 and associated guidelines.

To study changes in nociception and mRNA expression after SNI, 11–14 week old male 129/OlaHsd mice (Bristol University colony) were anesthetized, and the common peroneal and sural branches of the right sciatic nerve were sectioned leaving the tibial branch intact (Holmes et al., 2008). 7 days later mice were killed by cervical dislocation to obtain ipsilateral (SNI) and contralateral (control) lumbar L4 and L5 DRG pools each from 8 to 12 animals (*n* = 4). Tissues were frozen on dry ice and stored at − 80 °C.

Heterozygous mice with one targeted allele of Gpr151 were generated by Deltagen Inc. and obtained from the Jackson Laboratory (stock number 005805; strain B6.129P2-*Gpr151*^*tm1Dgen*^*/J*, i.e. targeted mutation 1, Deltagen), in which a portion of the Gpr151 coding region (nt 155–271 of NM_181543) was replaced by a SA-IRES-LacZ-Neo cassette, resulting in an allele with β-galactosidase expression under the control of the endogenous Gpr151 promoter and targeted disruption of the Gpr151 (Gpr151 MUT). All experiments were performed using 10–12 week old animals bred to homozygosity for the targeted allele and strain-, age- and sex-matched WT controls. Animals were genotyped by PCR with three primers:

Construct forward 5′-CTGGATCTCGAGTGATCAGGTACC-3′,

Gpr151 forward 5′- CCTAAACAAGAAGCTACCATCTGCA-3′,

Gpr151 reverse 5′-AGTCAGAGGACTTGCAGATGAACC-3′.

Cycling conditions were 95 °C for 7 mins; 40 cycles of 94 °C for 30 s/62 °C for 30 s/72 °C for 90 s; and a final 72 °C incubation for 10 min, resulting in amplified products of 178 and 395 bp from Gpr151 MUT and WT alleles, respectively.

### 5.2 Real-time quantitative RT-PCR assays

Total RNA isolation, DNase treatment and re-extraction, and reverse transcription (RT) reactions with random hexamers were as published (Kerr et al., 2004). Real-time RT-PCR assays and the endogenous control glyceraldehyde 3-phosphate dehydrogenase (Gapdh), GalR1 and GalR2 primer and probe sets were as reported (Hobson et al., 2006), except for the corrected GalR2 probe sequence 5′-TTCCTCACTATGCACGCCAGCAGC-3′ (mGalR2-46TAQ) used then and herein. The Gpr151 primer and probe set was designed using default parameters of Primer Express software (Applied Biosystems):

Forward primer 5′-GGGAACACGAAGGCCAAGA-3′,

Reverse primer 5′-AGTGGTACAAGTAAACACAGTAACGACAA-3′,

Non-extendable Taqman probe 5′-CTCAAGTCTGTTAATTGCAGCCCTCTGT-3′,

corresponding, respectively, to nucleotides (nt) 1273–1291, 1366–1338 and 1320–1293 of the reference RNA (NM_181543). The Gpr151 probe had a 5′ fluorescent reporter dye FAM (6-carboxy-fluorescein) and the 3′ quencher dye TAMRA (6-carboxy-tetramethyl-rhodamine), and assay primer and probe concentrations were optimized against partial-length Gpr151 cDNA cloned from mouse DRG (nt 982–1555 of NM_181543). All primers and probe sets were synthesized by Applied Biosystems. Relative mRNA expression levels were derived by the comparative threshold cycle (C_t_) method, with results presented as mean of log transformed data and statistical significance judged by two-tailed paired *t*-test, as previously described (Kerr et al., 2008). Each assay amplified a single product of the expected size, as detected by gel electrophoresis on 4% agarose gel (data not shown).

### 5.3 Pain-related behaviours

Mice were habituated to the testing chambers for 3 days for 30 min prior to testing. Paw thermal withdrawal thresholds were measured using a Hargreaves apparatus (Ugo Basile). Three measurements were taken for each hind paw. Paw withdrawal thresholds to mechanical stimuli were determined using Von Frey hairs employing the ‘up-down’ testing method as previously described (Holmes et al., 2003).

To determine inflammatory nociceptive responses the formalin test (Hobson et al., 2006) was used. 20 μl of 1% formalin was injected subcutaneously into the right hind paw and time spent in nocifensive behaviours (licking, biting, shaking, lifting of the paw) were measured in 5 min periods over 1 h.

For all tests the experimenter was blind to the mouse genotype.

### 5.4 Calcium flux assay

ND7/23 cells (ECACC 92090903, (Wood et al., 1990)) were transiently transfected with a Gpr151 plasmid (OriGene, USA) using Lipofectamine 2000 according to the manufacturer's instructions (Invitrogen), and maintained in normal culture medium overnight (high glucose DMEM with l-glutamine, supplemented with 10% FBS and 1% Pen/Strep, Sigma, UK) before being re-plated into dark walled 96 well plates (at a density of 7000 cells per well, resulting in 50–200 cells per field during the experiment). At the start of the imaging experiment, cells were washed once before loading with 2.5 μM Fluo-4 Ca^2 +^ indicator (Invitrogen) and 0.5 μM Hoechst nuclear stain in phenol red-free DMEM including 10% FBS, 100 U/ml penicillin, 0.1 mg/ml streptomycin and 25 mM HEPES (Sigma, UK), then incubated at 37 °C for 30 min. After loading the sample plate into IN Cell Analyzer System (GE Healthcare) and live cell populations imaged using × 10 objective magnification and various concentrations of porcine galanin (Bachem, UK) were added to experimental wells. Image analysis including localisation and quantification of fluorescence intensity, using the multi-target analysis algorithm was performed using IN Cell Analyzer work station 3.5 software (IN Cell Investigator, GE Healthcare). Green channel (Fluo-4) and blue channel (Hoechst) images were used to define whole-cell and nuclear regions, respectively. For graphical representation and statistical analysis the average Fluo-4 intracellular intensity (population-averaged) was calculated and standardised against the resting intracellular fluorescent intensity and expressed as relative fluorescence units (RFU).

### 5.5 Statistical analyses

Data are presented as the mean ± SEM. Quantitative real time RT-PCR data, baseline thermal withdrawal thresholds and cumulative formalin test data were analysed by Student's *t*-test. Mechanical withdrawal thresholds and formalin test data were analysed by two way repeated measures ANOVA. Results were taken to be significant if *P* < 0.05.

## Authors' contributions

FEH, NK and Y-JC undertook the experiments and statistical analysis; PV curated and genotyped the transgenic colony; CM provided technical assistance and access to equipment and FEH, NK, Y-JC and DW wrote the paper. All authors read and approved the final manuscript.

## Competing interests

All authors declare that they have no conflict of interest.
